# Working Women in Large Towns

**Published:** 1890-04-26

**Authors:** 


### Working Women in Large Towns.

### I.—IN ENGLAND AND IN AMERICA.

It has often been said, and said with truth, that the English people can never be persuaded to give serious attention to a subject until startled into action by some sudden and rude shock. Philanthropists and agitators alike must bide their time, no matter how important the questions they would raise may be. All in a moment the shock may come; the natural course of events, or even a lucky accident, may bring their subject to the top of the seething cauldron of the world's affairs; and then is their time to rush in, to catch the public's eye, and to get something done, or at least set going, while men are still smarting from the sudden shock. With regard to the question of women's work, however, we are not without hope that a succession of small shocks may supply the place of the greater one which will come some day if we refuse to open our eyes to the evils and dangers which are pressing hard upon our working women. The evidence given before the Sweating Commission, the Whitechapel murders, and the publication of such books as the "Bitter Cry of Outcast London," and Walter Besant's "Children of Gibeon"—all these must have made us think, and it will be well indeed if they make us do. To argue that the organization of industry must of necessity involve the presence of a certain unemployed margin, who will no doubt suffer acute distress, and that however much we may regret this, we can do nothing to prevent it, is weak and, we may add, criminal. Our first duty must be to study the forces at work in our midst, that we may see whether we cannot control and check them, or at any rate, set other forces in motion which shall prevent them from oppressing the weak and helpless, and bringing danger and disgrace upon the whole community.

And many as are the subjects which demand our attention at the present time, none, we think, is more urgent than this—the condition of working women in our large towns. For one thing, it is a large question. What their actual number may be, we cannot say; but the fact that there are eighty thousand more women than men in England and Wales is surely enough to set us thinking. Again, there are over two hundred thousand factory-girls in London alone, to say nothing of women working in shops, in private houses, and in their own homes. Evidently it is not a mere handful of people that we have to consider, but a very large, and it is to be feared, a quickly-increasing class.

Again, it is a highly important question. These working girls and women are or will be the mothers of a large part of the next generation; as they are, so will their children be, not merely by inheritance, but by home training and influence. And who can gauge the influence of women, not only over their children, but over their brothers, sweethearts, and husbands? "We might almost say," writes Miss Stanley, "that the welfare of the workgirl is at the root of the important questions now exercising the minds and thoughts of some of the best of our generation—the question, How are we to improve the lives of our working classes?"

Once more, it is a pressing—we might say, a terrible question. Women are weak; they lack not only the physical strength, but the nerve and the firm fibre of men; and in these days of fierce competition it is the weakest who goes to the wall. The Factory Acts are standing evidence that women have been found unable to protect themselves in the hard struggle for existence; and if this was the case some years ago, what must it be now, when week after week brings a fresh influx from the country into our over-populated towns, when crowds of foreign immigrants land at our ports, and even strong men begin to cry out in their distress for laws that shall protect them from each other—we might almost say from themselves?

America has recognized the importance and urgency of this question more promptly than we. Mr. Carroll D. Wright, Commissioner of Labour, has devoted a bulky volume to the consideration of the condition of American working women; and a very instructive volume it is. The agents of the department (mostly women) have fully investigated the condition of working women in twenty-two large cities; and elaborate tables are given, showing the average age at which women begin work, their health, their earnings, their expenses, their education and church attendance, their home and shop surroundings, and their character. This latter point being impossible to deal with satisfactorily by the statistical method, a short chapter is devoted to its consideration; and the Commissioner reports that "from all that can be learned, one need not hesitate in asserting that the working women of the country are as honest and virtuous as any class of our citizens."

From a useful topical analysis of the tables we learn that in the twenty-two cities in which inquiries have been made, the average earnings of working women (due allowance being made for "slack times") are 5 dols. 24 cents, or nearly 22s. per week, the concentration coming on earnings ranging from about 12s. to 28s. Of 13,822 women who reported their earnings, 373 earned less than two dollars per week, a very low wage when the cost of living is taken into account. That this is much greater than in England is evident. We should by no means speak, as does the Commissioner, of "women being willing to work from morning till night for the *paltry sum* of five dollars per week." Again, the report of an American charitable Home speaks of three dollars a week as being "expended on board, even at its lowest rate"; while Miss Stanley incidentally remarks in her "Clubs for Working Girls," that payments by the inmates of an English Home at the rate of 8s. 2d. per week, should cover the cost of board, if properly expended. On the whole, we should judge that the cost of living in an ordinary American town must be reckoned as at least half as much again as in an English town; and this, in effect, greatly reduces what seem to be the far higher wages paid in America. It is extremely difficult to arrive at any certain conclusions as to the relative prosperity of American and English working women, owing to the absence of sufficient data with regard to the latter, as well as to the different systems adopted by* investigators. So far as we are aware, the only estimates to hand as to the average wage for an Englishwoman's work are those of Miss Collet and Miss Black, one of whom puts it at twopence, the other at a penny, an hour; while, careful and minute as have been the accounts given by these ladies and others of the wages paid in various trades, we have no tables to compare with those of the American Commissioner, who gives the average amount earned in each trade, and in each branch of that trade, allowing in both cases for days lost. This being the case, comparisons are—if not odious, at least very difficult. In one instance, indeed, we have lighted upon particulars of an excellent English factory (where infants' and children's clothing is made) in which the work is said to be absolutely constant, and have compared the average wages earned there—fifteen shillings a-week—with those earned in a New York "Infants' and Children's Wear Factory." In the English factory the workers will earn thirty-nine pounds a-year; in the American, about sixty-nine—considerably more than half as much again, which, allowing for the American's expenses being half as much again as the Englishwoman's, will still leave her considerably better off. But in this, as in other cases which we have endeavoured to

* Mr. Giffen's comprehensive inquiry into the rate of wages generally will doubtless supply this want; at present, only that part of it relating to textile industries is completed, and with these (following the example of the American Commissioner) we do not in this series of articles propose to deal.

compare, the English data are too scanty to be definitely relied on.

On the whole, however, facts seem to point to the conclusion that the condition of American working women is better than that of their English sisters. In New York, where house accommodation is very bad, and foreign settlers are very numerous, there is doubtless much distress—though it does not seem to stagger and appal the investigator as does the distress in our metropolis. On the other hand, we read that in Chicago "even employments requiring no skill command pay enough to render girls independent, and that if displeased they leave on the slightest pretexts." As for Saint Paul, "no poverty among the working people is seen or heard of in the city," and many of the girls who have invested their little savings in a vacant lot of ground have by the rapid rise in the value of property acquired a modest competence. In Boston, again, we hear that "music, literature, art, lectures, are all within easy reach, and that the working-girls avail themselves of such privileges to a great extent"—a necktie-maker actually contributing excellent verses to a high-class magazine. This again goes to prove that the condition of American workers is fairly prosperous; for culture and refinement can never go with long hours and miserably paid work. As to "sweating," it appears to be a thing unknown. Of "home-work," which is not even alluded to, there can be but little, if any; for the Commissioner states that the working women of America are practically girls; and girls as a rule go out to work.

These considerations lead us to the conclusion that whether or no there is very much difference in the relative conditions of women who would be described in each country as "fairly paid," there is a much smaller amount of starvation wage and grinding poverty in America than in England. That this may long be so, or rather that the mother country may yet devise means for bringing the standard of comfort for her down-trodden workers up to that of America, must be the earnest hope of all those who have the cause of working women at heart, who believe with Mr. Booth* that one of the main conditions of human happiness is work, and are only anxious to see their sisters freed from the terrible concomitants of daily labour which have come to seem to many of us but natural and inevitable—long hours, starvation pay, squalid and unhealthy home and shop surroundings, helpless, hopeless drudgery—unescapable, alas! save at the terrible cost of self-respect and purity.

*We gladly take this opportunity of expressing our indebtedness to Mr. C. Booth, and his coadjutors, Miss Beatrice Potter, Mr. D. Schloss, and Miss Collet, whose invaluable book, *Life and Labour of the People*, should be in everybody's hands. From the first volume, *East London*, we have taken details of wages, etc., in several industries; and we would specially recommend to our readers Miss Collet's contribution on Women's Work, which is pregnant with thought as well as with information.

